# The Nervous System Orchestrates and Integrates Craniofacial Development: A Review

**DOI:** 10.3389/fphys.2016.00049

**Published:** 2016-02-19

**Authors:** Igor Adameyko, Kaj Fried

**Affiliations:** ^1^Department of Physiology and Pharmacology, Karolinska InstitutetStockholm, Sweden; ^2^Department of Molecular Neurosciences, Center of Brain Research, Medical University of ViennaVienna, Austria; ^3^Department of Neuroscience, Karolinska InstitutetStockholm, Sweden

**Keywords:** nervous system, craniofacial, development, glia, stem cell, tooth

## Abstract

Development of a head is a dazzlingly complex process: a number of distinct cellular sources including cranial ecto- and endoderm, mesoderm and neural crest contribute to facial and other structures. In the head, an extremely fine-tuned developmental coordination of CNS, peripheral neural components, sensory organs and a musculo-skeletal apparatus occurs, which provides protection and functional integration. The face can to a large extent be considered as an assembly of sensory systems encased and functionally fused with appendages represented by jaws. Here we review how the developing brain, neurogenic placodes and peripheral nerves influence the morphogenesis of surrounding tissues as a part of various general integrative processes in the head. The mechanisms of this impact, as we understand it now, span from the targeted release of the morphogens necessary for shaping to providing a niche for cellular sources required in later development. In this review we also discuss the most recent findings and ideas related to how peripheral nerves and nerve-associated cells contribute to craniofacial development, including teeth, during the post- neural crest period and potentially in regeneration.

## Integration of early neural derivatives with other cellular sources during craniofacial development

**Figure 1:**
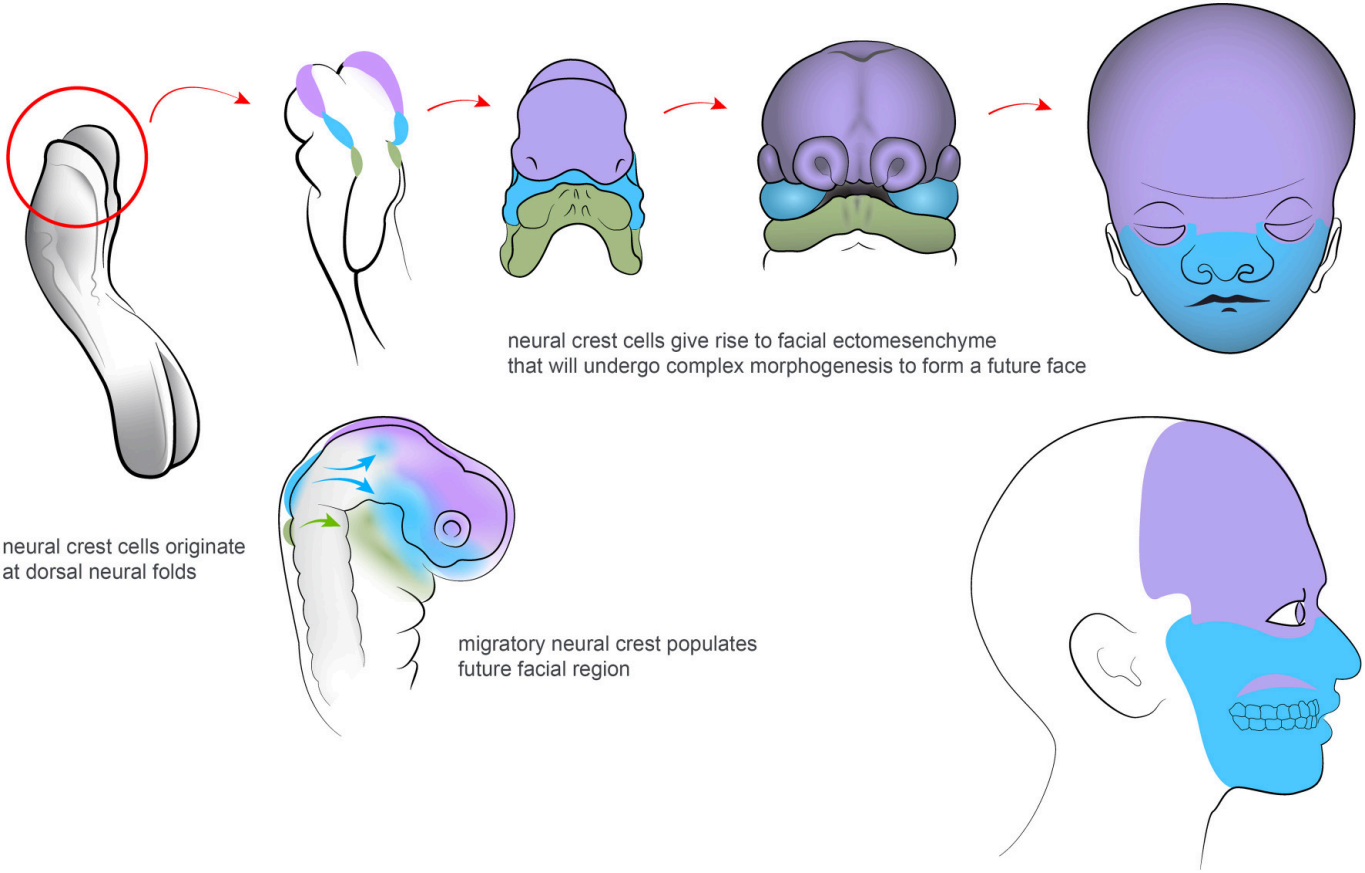
**Neural crest cells migrate from dorsal neuroectoderm and organize the facial development in vertebrate embryos**. Colors highlights regions of the developing and adult face that correspond to different neural crest populations along the posterior axis.

The involvement of neural structures in facial development begins as soon as Neural Crest cells (NCCs) emigrate from the dorsal neural tube (Simões-Costa and Bronner, 2015). The neural crest and its derivatives are keys to the understanding of the evolution and morphogenesis of the face (Green et al., 2015; see Figure 1). Thus, this transient cellular source contributes cartilage and bone, adipose tissue, tendons and fasciae, dermal fibroblasts and smooth muscles, pericytes, pigmentation, sensory neurons, peripheral glial cells and many more cell types to the developing head (Dupin et al., 2006). The vertebrate face is sculpted in a series of complex morphogenetic events. These involve neural crest coordination with other cellular sources such as mesoderm, endoderm and other ectodermal components (Couly et al., 2002; Rinon et al., 2007; Grenier et al., 2009; Marcucio et al., 2011; Van Ho et al., 2011).

More than 30 years ago, Gans and Northcutt proposed the hypothesis of “a new head.” This suggested that the evolutionary success of vertebrates is attributed to the development of their facial compartment. The face has emerged with various separated sensory organs due to a coordinated activity of the neural crest, together with other neural and skeletal elements (Gans and Northcutt, 1983). Historically, views and theories have held that the vertebrate head has its evolutionary origins in a segmented structure. This inferred that the head has evolved from of a series of complete head segments (metameres), such as those seen in lower species. However, there is no obvious scientific support for this view (Noden and Trainor, 2005). Instead, segmentation in the vertebrate head has been interpreted as the division of one or more embryonic tissues, such as the paraxial mesoderm, neurectoderm or endoderm into a series of iterative structures in the anterior to posterior axis (Noden and Trainor, 2005; Northcutt, 2008). During early embryogenesis, NCCs arise at the margins between the ectoderm (the future skin) and the neural plate. The formation of the neural crest is governed by a regulatory gene network, that will endow the NCCs with their typical features. This includes multipotency and migratory capabilities (Sauka-Spengler et al., 2007). After neurulation, NCCs from the roof plate of the neural tube undergo an epithelial to mesenchymal transition. They delaminate from the neuroepithelium and migrate outwards to populate the pharyngeal arches and other locations corresponding to the future face. NCCs also invade a number of specific sites in other parts of developing body.

Eventually, NCCs differentiate into a wide range of structures and tissues along the anterior-posterior axis of the embryo (Trainor, 2005; Gitton et al., 2010; Minoux and Rijli, 2010). Those NCCs that enter the future frontonasal prominence as well as the first and second pharyngeal arches will initiate, organize and coordinate craniofacial development. Having arrived at their destinations, the NCCs that are responsible for craniofacial morphogenesis are regulated by complex actions of the genetic machinery that will determine tissue patterning and cell differentiation. This involves signaling by e.g., FGF, Shh, Wnt, BMP, PDGF, retinoic acid (RA), endothelin and other molecules (Kurihara et al., 1995; Clouthier et al., 2003; Macatee et al., 2003; Abzhanov and Tabin, 2004; Jiang et al., 2006; Abe et al., 2008). NCCs will mainly form the anterior compartment of the head, while the posterior part, including the cranial roof is created largely by mesoderm (see Noden and Trainor, 2005). The relative size and shape of the anterior neural tube probably plays an important role in facial shaping. Thus, these parameters, together with local physical forces, may influence the amount and spatial distribution of emigrating NCCs during early development. The logics of setting down the boundary between neural crest and mesoderm-derived parts may also be important for differences in bone geometry and facial modeling. Furthermore, it might be relevant for the evolutionary plasticity of a head in multiple vertebrate species (Gross and Hanken, 2008).

Coordination and integration are key features during both early and late head and neck development. For example, induction of tracheal cartilages as well as some skeletal components of upper face and jaws depends on the activity of the endoderm and Wnt signaling pathways (Couly et al., 2002; Snowball et al., 2015). Dental placodal epithelia influence the competence of underlying mesenchyme while producing teeth and vice versa (Thesleff, 2003).

One of the most striking concerted event during facial development is when the Frontonasal Ectodermal Zone (FEZ; Hu et al., 2003; Hu and Marcucio, 2009a), a specific region in the anterior facial ectoderm, regulates the behavior of ectomesenchymal cells and skeletogenesis. This occurs via regionalized secretion of various morphogens, including Shh, Fgf8, and BMPs. FEZ may be considered as a facial organizer, as evidenced by the fact that transplantation or rotation of FEZ causes ectopic formation of skeletal elements or altered dorso-ventral axis in the forming face, respectively; (see Marcucio et al., 2011) for in-depth discussion. On the other hand, the competence to create a particular, species-specific facial morphology in response to epithelial signals is attributed to the neural crest. Transplantation experiments have clearly demonstrated that the final facial shape in a host embryo is determined by cranial neural crest of the donor (Helms and Schneider, 2003; Schneider and Helms, 2003). In general, numerous studies converge on the possibility that NCCs, after their immigration from the anterior dorsal neural tube, coordinate with FEZ formation to produce the outline of the face (Marcucio et al., 2011). Not only skeletal elements become transformed according to the instructive cues and typical morphology of the donor neural crest. The facial muscles also change accordingly, although the skeletal muscles in the developing face are derived from the mesoderm of the host (Tokita and Schneider, 2009). Hence, NCCs pattern developing head muscles (Rinon et al., 2007).

The instructive competence of the neural crest is further illustrated by experiments where mouse neural crest was transplanted into chick embryos, which led to the formation of dental primordia in the developing jaws (Mitsiadis et al., 2003). This shows that mouse neural crest can unlock the potential of chick ectoderm to form teeth. Similarly, Eames and Schneider demonstrated that neural crest transplantations between duck and quail change the pattern of cranial feathers according to the donor's profile. This includes expression of key morphogens in developing feather placodes (Eames and Schneider, 2005). These experiments provide critical examples of the shared competence and intricate interplay between epithelium and underlying neural crest-derived mesenchyme in the complex morphogenetic events that occur in a forming head.

Being derived from neural ectoderm, NCCs signal back to the developing CNS and modulate Fgf8 expression in the Anterior Neural Ridge and the isthmus in the brain. Moreover, Foxg1 expression, which is vital for fore- and midbrain patterning, is regulated by Smad1 activity of NCCs (Creuzet et al., 2006; Le Douarin et al., 2007; Creuzet, 2009a,b; Aguiar et al., 2014). Hence, the neural crest acts as an important signaling center that controls brain development (Le Douarin et al., 2012).

Disturbances in the normal migration and subsequent actions of NCCs and other inductive cell sources may cause abnormal development, as seen in a number of human syndromes with craniofacial malformations (Noden and Trainor, 2005; Chai and Maxson, 2006; Walker and Trainor, 2006). Among those most commonly observed are congenital disorders of the lip and/or palate, but a range of other dysfunctions in the soft and mineralized tissues of the head and face are caused by genetic deviations. For obvious reasons, an understanding of the etiology of these aberrations is fundamental for basic and translational science. Thus, novel data of this kind may subsequently be applied in the clinic to treat or even prevent craniofacial congenital malformations.

Interestingly, recent studies have clearly demonstrated that the developing neural structures do not cease to influence the morphogenesis of other cranial tissues and organs after NCCs have finished their migration from the dorsal neural tube. A large body of evidence now points toward the fact that the brain, the neurogenic placodes as well as the peripheral nerves are important for correct facial shaping as well as for providing necessary cell types to multiple locations in the entire head, until late developmental stages or even in adulthood.

### Further reading:

1. Molecular mechanisms of cranial NCCs migration and patterning in craniofacial development (Minoux and Rijli, 2010).
2. Neural crest and the origin of vertebrates: a new head (Gans and Northcutt, 1983).
3. Establishing neural crest identity: a gene regulatory recipe (Simões-Costa and Bronner, 2015).
4. Evolution of vertebrates as viewed from the crest (Green et al., 2015).

## Brain-dependent integration in the cranial compartment

At post-neural crest developmental stages, the growing brain continues to play an important role in coordinating and assisting the development of other craniofacial parts and tissue types. In the past, numerous suggestions have been made regarding the developmental interaction between the brain and the facial compartment. Physical interactions with underlying mechanical forces should be of at least some significance in the coordination of brain and face morphogenesis and growth. Indeed, many cases of microcephaly or macrocephaly are associated with changes, often dramatic, in the function and shape of the facial compartment (Kivitie-Kallio and Norio, 2001; Chen et al., 2004; Vasudevan et al., 2005). Human trisomy may involve severe disturbances in the patterning of the skull base, accompanied by disruptions in cranial nerve development. This underpins the fact that knowledge of complex osteogenic–neural dynamics is integral for the understanding of the pathophysiology behind genetic craniofacial malformations (Demyer et al., 1964; Colleran et al., 2014; Reid et al., 2015). Pushing this line of reasoning further, one can speculate that the nervous system might and should be capable of influencing the size of its protective skeletal encasement with corresponding facial compartment. Such a developmental integration would have emerged over millennia of co-evolution, leading to a sophisticated brain-to-skeleton crosstalk. This, in turn, would allow for a coordinated volume increase over developmental or evolutionary time. This concept is not new and appears multiple times in the literature. Biegert and De Beer were among those that propagated the notion that increasing brain size would cause predictable changes in the cranium (De Beer, 1937; Biegert, 1963). Indeed, Hallgrimsson and co-authors demonstrated that experimentally induced changes in brain size co-vary with alterations in morphologies of the skull base. This is in agreement with predictions of Biegert's model (Hallgrímsson et al., 2007; Hallgrímsson and Lieberman, 2008; Lieberman et al., 2008). For a detailed discussion of the “spatial packing” hypothesis, the brain as the architectural foundation of the face and other relevant issues including synchronized changes in skull base, please see the review by Marcucio et al. (2011).

The mechanisms that mediate the coordination between the growing brain and its surrounding cartilage and bone may include so called “quasi-static strain” tension stress, and intracellular systems of tension sensing and response via proliferation and production of the extracellular matrix (Moss and Young, 1960; Henderson et al., 2005; Jaalouk and Lammerding, 2009; Temiyasathit and Jacobs, 2010). For an extensive discussion of cranial bone development matching brain growth (see Richtsmeier and Flaherty, 2013).

**Figure 2:**
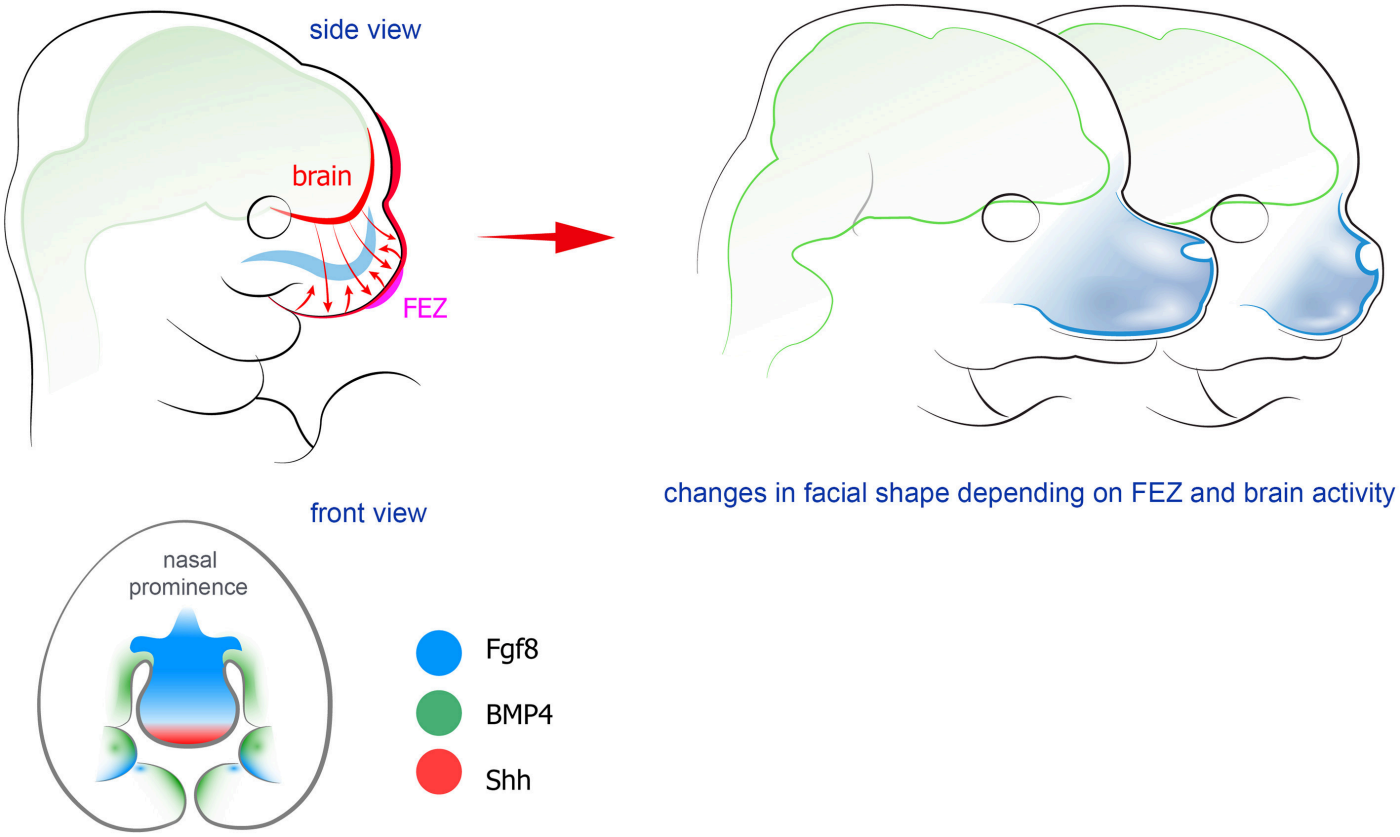
**Forebrain and FEZ exchange signals to shape the facial compartment**. Factors produced by the epithelium in the frontal face (FEZ) control the formation of future skeletal elements together with signals coming from the developing forebrain. Manipulation of FEZ leads to changes in the shape of **Figure 5**. Peripheral nerves host and transport neural crest-derived associated glial cells that give rise to a number of cell types in developing body. Left: examples and position of nerve-derived cell types. A significant number of melanocytes (purple circles on a scheme) in a head-neck region is produced from recruited Schwann cell precursors. Additionally, some mesenchymal progenitors in developing teeth (shown as red ellipses) are also recruited from the peripheral nerves. At the same time all parasympathetic neurons in the head are derived from nerve-associated cells. Right: the spectrum of currently confirmed nerve-derived cell types in the developing face (right panel).

As mentioned above, physical interaction and integration in the developing head have been topics for research and discussion since long. However, more recently a signaling crosstalk between the developing brain and the face was identified. This crosstalk commences early, when NCCs influence the ongoing development of the anterior CNS in the embryo (Creuzet et al., 2006; Le Douarin et al., 2007; Creuzet, 2009a,b). It became obvious that Shh-based signaling coordinates the development of the brain and the face. It has been widely known that mutations in the Shh pathway cause a number of pathologies involving both facial and brain-related phenotypes (holoprosencephaly, Greig Cephalopolysyndactyly, Gorlin syndrome; Ming et al., 1998; Pan et al., 2013; Chaudhary et al., 2015). In line with this, it was recently shown that silencing of Shh signaling in the brain influences Shh expression in FEZ, and causes phenotypes similar to holoprosencephaly (Chong et al., 2012; see Figure 2). Further studies from the Hallgrimsson and the Marcucio laboratories established that a Shh-responsive signaling center in the anterior CNS controls facial development through actions on the FEZ organizer in the anterior facial ectoderm (Hu and Marcucio, 2009b). Experimental disruption of Shh in the brain distorted facial development, but it was possible to rescue facial shape to a large extent by early application of Shh to the embryos (Chong et al., 2012). Further studies also highlighted an important evodevo aspect: animals with different facial morphology demonstrate significant differences in the organization of Shh-releasing zones in the face and in the forebrain (Hu et al., 2015). For example, the spatial structure of FEZ is clearly different between mammals and birds. Moreover, it is possible to interfere and change that structure in chick embryos. This yields chick embryonic faces that bear resemblance to faces of mice embryos (Hu and Marcucio, 2009b). In addition, both FEZ and Shh-releasing regions in the forebrain appear to be different between avian species. Thus, when duck forebrain was transplanted into the chick embryo, the facial compartment started to develop according to the donor tissue (Hu et al., 2015; Smith et al., 2015). For a detailed discussion regarding the molecular dialogue between brain, FEZ and facial compartments (see Marcucio et al., 2011, 2015; Young et al., 2014).

The development of the pituitary gland, an endocrine gland with modular structure, provides another example of a complex molecular dialogue between the brain and oral epithelium based on Shh, Fgf8, and BMP4 signaling. The pituitary gland originates in part from the most anterior neural ridge in the developing brain and in part from Rathke's pouch. Rathke's pouch formation begins as an invagination of oral ectoderm, which is initiated in response to BMP4 provided by the ventral diencephalon. Shh also plays a prominent role in this process (Zhu et al., 2007). The gland starts to develop prior to the formation of the surrounding *sella turcica*, a depression in the sphenoid bone. Some pathological cases related to hypo- or hyperthyroidism show that variations in size of the gland is reflected in the shape and volume of corresponding lacunae in *sella turcica* (Gopalakrishnan et al., 2015). This suggests the presence of at least a mechanical coordination in the development of the pituitary gland and the skeletal compartment that enclose it.

The eye is a prominent craniofacial organ derived from the developing brain. From an evolutionary standpoint, photosensory systems are products of neuroepithelium from different parts of embryonic or larval CNS. This explains the fact that the eyes or ocelli are located inside of the nervous system in a number of lower animals (Ivashkin and Adameyko, 2013). Ontogenetically and phylogenetically, the neural folds in what eventually will become the diencephalon form indentations, and these expand to create the optic vesicles and eventually the optic cups. The lens placode, the origin of the lens of the eye, arises from the extended anterior placodal area (Toro and Varga, 2007) and invaginates adjacent to the optic cup. Brain-derived optic vesicle interacts with future lens tissue to promote maturation and further transformations (signaling crosstalk in eye development is reviewed in Gunhaga (2011). Later on the lens cells will differentiate, elongate and become transparent. The outer and inner sheaths of the optic cup differentiate into the pigment and neural retinas, respectively. This process is, meanwhile, strictly coordinated with the formation and insertion of the extraocular muscles that surround the optic cup. This is indicated by observations of frequent anomalies of the extraocular muscles in patients with rather extreme craniofacial malformations. For example, in human fetuses with anencephaly or other grave skull deficits, absence or abnormalities of the extraocular muscles were found, including anomalous insertions. Similar feature may be present also in milder developmental disturbances of the craniofacial region (Plock et al., 2007).

**Figure 3:**
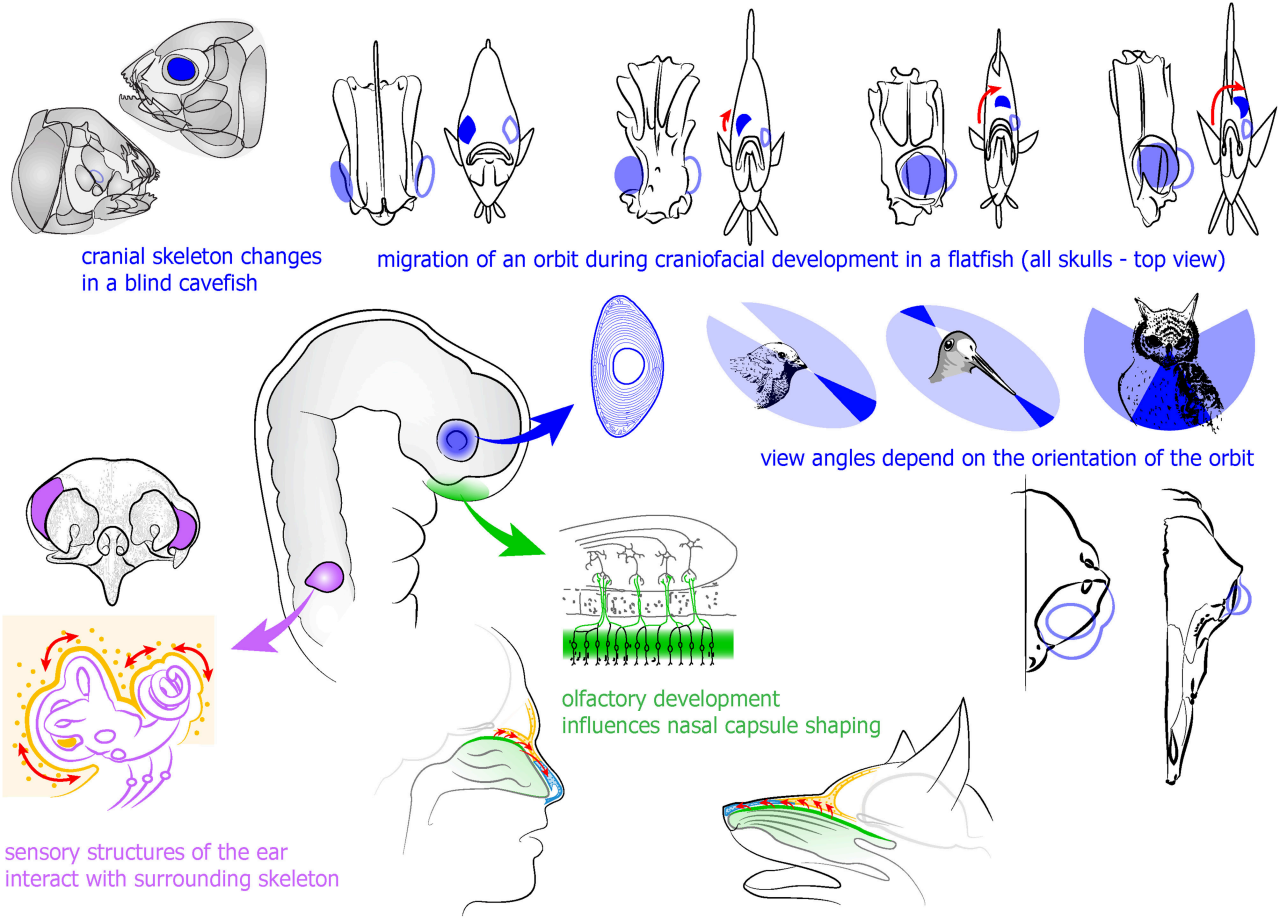
**Developing eye and sensory placode-derived structures influence the development of surrounding skeletal and other tissues in the head**. Upper part (in blue): developing eye influences the formation of surrounding skeleton as evidenced in blind cavefish and observed in the development of a flatfish. Integration of an eye into orbit provides the specie-specific view angle and stands behind stereoscopic vision. Left (in purple): otic vesicle and sensory ear components induce and integrate surrounding cartilage and bone. Lower part (in green): olfactory epithelium development influences formation of surrounding skeletal parts.

Little is known about the integration of the eye and its muscles into the developing skeletal socket of the face, the orbit. The orbit is created through intramembranous ossification of different embryological structures that eventually will form seven bones. The concomitant incorporation of the visual organ into the orbit during this process must require an extremely complex synchronized mechanism. Eyes are sensory organs of utmost importance, and their position in a head, their direction and relative size are tightly selected by evolution in all animal species. This, in turn, depends on the evolutionary and ontogenetic interactions of the eyes with the forming cranium. Changes in orbit position and orientation, for example, enabled binocular vision—one of the factors that shaped human face (see Figure 3). This implicates that disturbances in craniofacial bone development might be accompanied by deficits in the eye or its appendages. It has been suggested that the developing eye, in addition to the forebrain and FEZ, is an important organizer of craniofacial development, including roles non-related to visual perception (Kish et al., 2011). Thus, the eye vesicle influences neural crest migration (Langenberg et al., 2008) as well as production of retinoic acid (Matt et al., 2005, 2008; White and Schilling, 2008) and Shh. Indeed, it is possible that eye-derived retinoic acid or Shh mediate a crosstalk with mesenchymal tissues of the future orbit, coordinate patterning of extraocular muscles and influence the geometry of periocular and other skeletal parts. There are no animals where eye development programs are completely aborted. All eyeless species of fish and salamander have early steps of eye development, which could well be necessary for proper integration of structures during craniofacial morphogenesis (personal communication William R. Jeffery, University of Maryland, as described in Kish et al., 2011).

The migrating orbits in developing flatfishes represent one of the best examples of coordination between orbit position, cranial bone structure and the eye (see Figure 3). In these fishes, one eye moves to the contralateral side as an adaptation to the lifestyle at the sea floor. The movement of an orbit is caused by proliferating periocular cell masses on the future ventral side of the head. The rotation of the cranial bones depends on the translocation of the eye. Inhibition of proliferation in the suborbital area causes an abrogated migration of the eye to the opposite side, and a changed structure of a skull (Bao et al., 2011). In line with this, the cave-dwelling fish *Astyanax mexicanus* demonstrates a variety of eye-dependent transformations of the cranium in different subspecies living in conditions with or without light. In addition, transplantations of embryonic lenses and removal of optic vesicles in *Astyanax* showed that six suborbital bones and cranial neuromasts partially depend on the presence of an eye (Yamamoto et al., 2003). The fact that it is possible to change the cranial bone size and geometry by manipulating eye development is indicative of a close combination and crosstalk between the developing eye and the skull during ontogeny. The nature of this crosstalk, though, is still not understood. Interestingly, in many groups of animals there are cartilages and bones that form what is called an ocular skeleton directly inside of the eyes. The ocular skeleton may include a ring of scleral ossicles, and a cartilage that supports the eyeball or join the ossicles together. The function of these skeletal elements that often attain a significant size is not clear (Franz-Odendaal, 2011).

Taken together, it seems that signaling crosstalk between the developing brain (including parts forming the pituitary gland and eyes) and surrounding tissues provides both for a coordinated general shaping of the facial compartment and for proper development of particular structures.

### Further reading:

1. Mechanisms that underlie co-variation of the brain and face (Marcucio et al., 2011).
2. Embryonic bauplans and the developmental origins of facial diversity and constraint (Young et al., 2014).
3. The eye as an organizer of craniofacial development (Kish et al., 2011).

## Sensory neurogenic placodes in the cranial compartment

Cranial neurogenic placodes originate as ectodermal thickenings from the anterior neural plate border area, the so-called pre-placodal region (Streit, 2007). Neural plate and mesoderm-derived FGFs act to induce the pre-placodal region concomitant with a local downregulation of BMPs and Wnts (Litsiou et al., 2005; Streit, 2007; Park and Saint-Jeannet, 2008; Grocott et al., 2012; Groves and LaBonne, 2014). Placodes contain clusters of progenitor cells that will give rise to an array of sensory and neurosecretory organs. With the exception of the nodose ganglia, the placode-derived structures occupy specifically designed skeletal lodges in the cranium. Some evidence point toward the fact that all neural placodes share a common evolutionary origin. This ancient origin was subsequently elaborated in different ways to produce a number of tools that are specified to gather information about the environment (Schlosser, 2015). From an evolutionary point of view, this would have enabled a profound change in lifestyle, from a passive feeding to an active search and hunt of prey (Lassiter et al., 2014; Schlosser, 2014). Such a dramatic change would inevitably cause modifications of the feeding system with further development of jaws, and also improve the protective encasements for CNS and sensory organs. However, the mechanisms behind the sophisticated interactive coordination during craniofacial sensory and skeletal co-evolution are not fully understood.

The otic placode is induced by extrinsic FGF signals from the pre-placodal region immediately bordering the anterior neural plate (Ohyama et al., 2007). It later develops into the inner ear, which eventually becomes enclosed in the densely mineralized petrous part of the temporal bone (Basch et al., 2016). The inner ear is connected to the other sections of the auditory system, the middle and the outer ear. These parts have different developmental origins. The middle ear is separated from the auditory canal by the tympanic membrane, which has an outer layer continuous with the ectoderm of the ear canal, and an inner layer that is assumed to be of endodermal origin.

The middle ear is a cavity in the temporal bone with a very complex anatomy. It is covered by endoderm- and neural crest-derived epithelium (Thompson and Tucker, 2013) and is bridged by the three middle ear ossicles, malleus, incus, and stapes, which are the result of endochondral ossification from neural crest of the first and second arches (reviewed by Anthwal and Thompson, 2016). The pinna, which gives rise to the auricle, is discerned as six small mounds, or hillocks, derived from the 1st and 2nd pharyngeal arches at E11.5 in the mouse, and 6 weeks gestation in humans. These hillocks grow and unite to form a cartilage-filled structure at the entrance of the auditory canal (reviewed by Anthwal and Thompson, 2016). The nature of organization between neural, glia or sensory cells and other different types of tissues during ear ontogeny is not well understood. It may be suggested that extrinsic co-regulation and active crosstalk between tissue types might not be necessary during development of an ear, since coordinated morphogenesis can be achieved due to autonomous behavior of every partaking tissue after millennia of a tight co-evolution. When considering the evolutionary history of the middle and outer ear in vertebrates, there will be some significant support for such logics, especially taking into account significant variations in middle ear structure without accompanying changes in the basic configuration of the inner ear (Maier and Ruf, 2015). However, despite possible autonomic control and congruence of different parts of a hearing system after a protracted co-evolution, there are clear examples that demonstrate a functional cross-talk between placode-derived and other structures that shape the hearing sensory organ. In the beginning of the twentieth century, Luther demonstrated that surgical removal of the otic capsule from frog embryos prevents formation of otic cartilage. However, this operation did not affect columella auris. Grafting of the otic vesicle into a head region could induce formation of surrounding cartilage, but this did not occur in cases of transplantations into the trunk (Luther, 1924). On the other hand, in mice with a conditional knockout of Dicer1, all major cranial sensory organs are severely impaired and a craniofacial phenotype occurs. These mice demonstrate a truncated development of the otic capsule and some changes in the craniofacial development associated with the loss of olfactory epithelium and forebrain (Kersigo et al., 2011). In agreement with this, Six1 knockout embryos with abnormally developed placode-derived sensory components show distorted cochlea morphologies (Laclef et al., 2003).

The olfactory and the vomeronasal epithelia are generated by the olfactory placode, and line bony skull parts of impressive sizes in many animal species with a highly developed sense of olfaction. It appears as if the geometry and the amount of necessary bone are proportional to the surface of olfactory epithelium in various animals. Moreover, the cribriform plate appears to have a size and structure that corresponds to the olfactory needs (Bird et al., 2014; Van Valkenburgh et al., 2014). In humans, with a poor sense of smell in comparison with for example dogs, the corresponding cranium parts are proportionally much smaller. The protracted co-evolution of skeletal and mesenchymal structures in the anterior head allowed a fusion of olfactory and respiratory passages (Jankowski, 2011). However, similar to what was discussed above, a perfect fit of various tissues in the nose can be achieved through evolutionary autonomic tuning of developmental dynamics. So far there is no clearly identified regulatory crosstalk between skeletal tissues and the olfactory epithelium that they host. However, more than 70 years ago, Schmalhausen demonstrated that the formation of the cartilaginous nasal capsule depends on olfactory epithelium development, using surgical methods in urodeles (Schmalhausen, 1939). Furthermore, Burr and Reiss showed the response of the facial skeleton after removal of the nasal placode (Burr, 1916; Reiss, 1998). In line with this, analyses of Six1 and conditional Dicer1 knockout animals indicated some degree of extrinsic signaling and synchronization, since the affected olfactory placodes in these animals yielded disturbances in facial shape (Laclef et al., 2003; Kersigo et al., 2011). It seems reasonable to assume that neural placode-derived structures destined to become e.g. olfactory or auditory or vestibular, interact with the cellular progenies that eventually will form their protective cartilage and bony cases, see Figure 3. Despite this, the detailed coordination of sensory organ and cartilage and bone formation in the head, in terms of molecular signaling, does not seem to have been the subject of much attention.

The ophthalmic and maxillary placode of the trigeminal nerve contributes sensory neurons to the trigeminal ganglion (see Schlosser, 2010). These cells, which coalesce with neural crest-derived neurons, convey sensory modalities such as e.g., touch, temperature and pain, from the exterior and interior of the head. The trigeminal ganglion, which harbors the trigeminal primary sensory neurons, is situated in a tightly sculpted bony furrow of the sphenoid bone in the middle cranial fossa. With regard to the sensory innervation of the facial mesenchymal derivatives and especially bone, not much is known to what extent it might convey secreted signals to affect developmental events there. However, the opposite is true: development of craniofacial non-neural structures influences the gene expression in trigeminal neurons. For instance, target-derived BMP4 helps to determine the phenotype of trigeminal ganglion nerve cells in mice (Hodge et al., 2007). In this way, neural crest- and placode-derived nervous system progenitors on the one hand, and ectomesenchymal ones on the other, adapt very different fates but then collaborate to control the ontogeny and evolution of the different systems they eventually will form.

### Further reading:

1. Vertebrate cranial placodes as evolutionary innovations—the ancestor's tale (Schlosser, 2015).
2. Where hearing starts: the development of the mammalian cochlea (Basch et al., 2016).
3. The development of the mammalian outer and middle ear (Anthwal and Thompson, 2016).
4. Revisiting human nose anatomy: phylogenic and ontogenic perspectives (Jankowski, 2011).

## Nerve-organogenesis interplay in the cranial compartment

**Figure 4:**
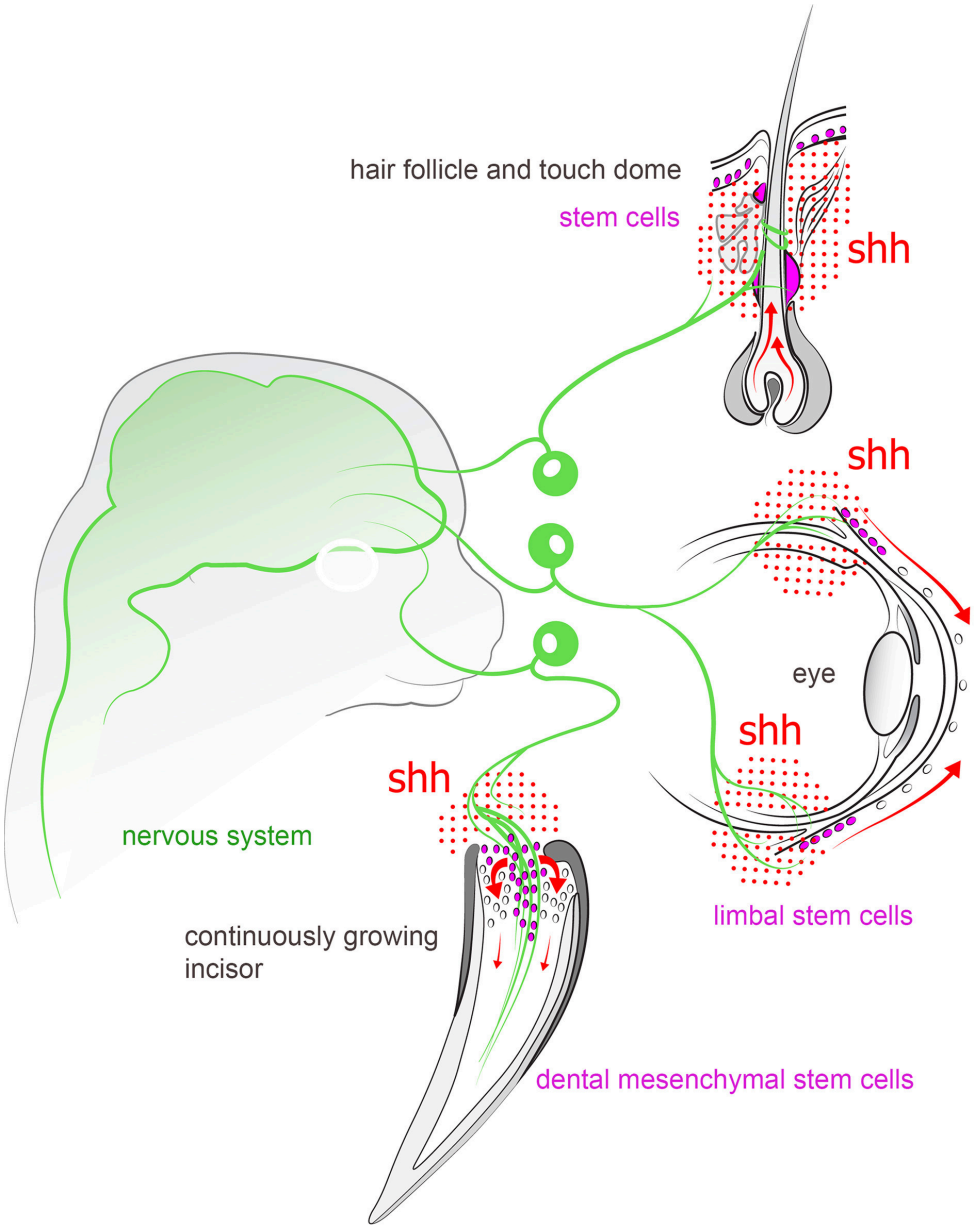
**Peripheral sensory nerves inject Shh into variety of structures in the head and tune the activity of stem cell compartments**. For instance, nerve-derived Shh plays an important role in controlling stem cell niches in continuously growing incisor, hair follicle, touch dome, and limbal region of cornea in the eye.

Recently it became apparent that peripheral nerves play a number of non-canonical functions related to morphogenesis of innervated tissue, in addition to canonical action potential propagation (Kaucká and Adameyko, 2014). A striking example where peripheral innervation seems indispensable for proper tissue or organ generation/regeneration in vertebrates is provided by fish teeth (Tuisku and Hildebrand, 1994). Cichlids were used to perform unilateral denervation studies of the lower jaw, followed by extraction of mandibular teeth. Corresponding teeth were extracted from both sides of the jaw, and the unoperated side served as control for the denervated side. Cichlids have continuous tooth replacement under normal circumstances. Interestingly, after a one-year follow-up, it was found that the teeth on the side where the nerve was lesioned did not regenerate, in contrast to the control side with the intact nerve. This is a strong indication that there is a link between peripheral innervation and organ formation. Hypothetically, nerves may provide signals for tooth formation by presenting factors that influence the ectomesenchyme or dental epithelium. Organotypic *in vitro* and *ex vivo* cultures have shown that tooth development can proceed without nerve fibers (Lumsden and Buchanan, 1986). Tooth germs can develop in standard tissue culture conditions for 7–8 days, but eventually become flattened and disorganized. When using 3D culture systems, this period can be slightly extended (Sun et al., 2014). However, tooth explant cultures have been performed with tissues that may already be committed to a dental fate, and it cannot be altogether excluded that the actual initial tooth-determining signals to epithelium and mesenchyme are of neural or glial origin. Indeed, a recent study from Yang Chai's laboratory demonstrated that nerve-dependent cues are necessary for driving the activity in a mesenchymal stem cell niche associated with the neuro-vascular bundle in continuously growing mouse incisors. Thus, Shh released from trigeminal ganglion sensory endings proximal to Gli1^+^ dental mesenchymal stem cells (MSCs) appears to be a key factor to ensure continuous tooth growth. Ablation of the sensory innervation of the incisor eventually led to the deterioration of the tooth (Zhao et al., 2014; see Figure 4).

The taste bud is another craniofacial sensory organ whose fate is intertwined with its innervation. Earlier transplantation studies in the axolotl have suggested that nerve fibers may not be necessary *per se* for the primary taste bud development, since very early donor embryo oropharyngeal tissue devoid innervation as well as taste buds develop taste buds when grafted to the trunk of a host embryo (Barlow et al., 1996). However, during subsequent development, it is since long well established that taste bud growth and maintenance is dependent on an intact innervation, originating from the VIIth and IXth cranial nerves (Oakley and Witt, 2004). The molecular regulation behind this phenomenon has been linked to the expression of Shh in taste bud basal cells. After chorda tympani denervation, taste buds appear reduced in size as compared to control side of the tongue (Li et al., 2015). Furthermore, long term application of the Shh inhibitor LDE225 results in a similar phenotype, with a severe size reduction of taste buds (Kumari et al., 2015). Shh expression is lost after denervation, which would explain why progenitor activity is hampered (Miura et al., 2006). Furthermore, artificially induced expression of Shh in lingual epithelium induces the formation of ectopic taste buds, which in contrast to normal taste buds seem to be independent of individual innervation (Castillo et al., 2014). Taken together, these data indicate that nerve-derived Shh maintains taste buds. Thus, neural Shh may control proliferation of adjacent progenitors, and is crucial for the regulation of taste bud density on the tongue. In addition to this coordinated development of neural and an epithelial sensory system, recent data indicate a common genetic ancestry between the gustatory system and teeth. In teleosts (cichlids), oral epithelium seems to possess an ancient inherent single regulatory gene system. This system, which may persist also in mammals, is suggested to be capable of co-patterning teeth and taste buds. Specifically, Wnt determines density (coordination) of teeth and taste buds, while BMP and Shh influence organ characteristics (Bloomquist et al., 2015). If, and if so to what extent this genetic pathway would be under the developmental influence of the ubiquitous innervation of the oral cavity has however not been addressed.

The touch dome (Merkel cell-neurite complex), a highly innervated epithelium-derived sensory structure in the skin, also depends on neural hedgehog signaling for self-renewal. Denervation experiments or ablations of Shh in sensory neurons showed that touch dome stem cells fully rely on the neural environment for their propagation and maintenance. Accordingly, an elevated production of Shh results in neoplastic proliferation of touch dome cells (Xiao et al., 2015). In line with this, Shh appears to be an important signal for some particular Gli1^+^ hair follicle epithelial cells in the bulge region. Gli1^+^ bulge cells are known to incorporate into healing skin lesions, and also to function as stem cells to restore hair follicles at each anagen phase (Brownell et al., 2011).

Another example of a nerve-dependent stem cell compartment in the face is represented by limbal stem cells in the cornea of the eye. Neurotrophic keratopathy (NK) is a disease manifested by a progressive corneal degeneration that is the result of corneal nerve dysfunction. Multiple defects eventually develop in the cornea, including perforations and corneal thinning. A recent study identified the relationship between corneolimbal epithelial stem cells and sensory nerves with the help of a mouse denervation model. The authors of this study electro-coagulated the ophthalmic branch of the trigeminal nerve, and after 7 days they observed a significant loss of corneolimbal stem cell markers as well as a reduction in the colony-forming efficiency of stem cells obtained from denervated corneas (Ueno et al., 2012).

The salivary gland has proved to be a good model system to address how local organogenesis can be directed by the peripheral nervous system. Sarah Knox and co-workers discovered that parasympathetic neurons that innervate salivary gland also control the organogenesis of the developing gland. Their studies demonstrated that during early salivary gland development, parasympathetic axons grow to surround the end buds of the ductal epithelium. Once there, vasoactive intestinal peptide (VIP) is being released from nerve endings and diffuses toward the epithelium. VIP then activates the cyclic AMP (cAMP)/protein kinase A (PKA) pathway, and helps to increase duct elongation and the subsequent formation of a single contiguous structure. Nerve depletion during this process causes a distorted tubulogenesis. When the lumen is established, VIP is also required for its enlargement through a cAMP/PKA/cystic fibrosis transmembrane conductance regulator (CFTR)-dependent pathway (Nedvetsky et al., 2014). This is *prima faciei* evidence for the necessity of an intact peripheral nervous system for correct craniofacial organ development, and underlines the need for more research into developmental interactions between nerves and other epithelial derivatives. For example, postganglionic sympathetic neurons that express a cholinergic/noradrenergic co-phenotype are present before the sweat glands that they will innervate are formed (Schütz et al., 2008). Thus, it is possible that these nerve fibers may influence target organ formation in an as yet unknown manner.

Diseases causing abnormal cranial nerve development often lead to aberrant growth of target organs and tissues. It is evident that the peripheral innervation in many cases is needed for proper development and function of orofacial structures (Pagella et al., 2014). The aetiologies behind congenital facial abnormalities vary. They can be caused by genetic or, most commonly, unknown factors. Deformations in facial growth can be environmentally influenced during embryogenesis and disruptions in the growth can also have metabolic, vascular and/or teratogenic causes. One example is Möbius syndrome that causes palsy and nerve weakness, which in turn gives facial asymmetry. This congenital disease is characterized by deficient innervation (abducens (VI) and facial (VII) nerves), and causes clinical symptoms such as deafness, tooth anomalies, and cleft palate (Rizos et al., 1998). Another example is hereditary sensory and autonomic neuropathy type IV (HSAN IV), a rare inherited disorder of the peripheral nervous system. It is the caused by mutations in the neurotrophic tyrosine kinase receptor 1 gene (*NTRK1*), which encodes the high-affinity nerve growth factor receptor TRKA. Affected patients of this multisystem syndrome have a severe peripheral nerve fiber loss and display a lack of reaction to pain stimuli, inability to sweat, and mental retardation. Interestingly, from a developmental point of view, oral and craniofacial manifestations include missing teeth, nasal malformation, cleft palate, dental caries and malocclusion (Gao et al., 2013). Furthermore, severe hemifacial atrophy is seen in Parry-Romberg syndrome. This disorder, which may be accompanied by a range of neurological pathologies, including trigeminal neuralgia, has an unclear etiology but has been linked to defects in innervation (Vix et al., 2015). Various problems in the cervical sympathetic trunk leading to dysfunctions in the sympathetic system seem to play important roles in the development of facial atrophy, and cause a number of additional symptoms including alopecia and scleroderma (Scope et al., 2004) Indeed, experimental animals with ablated superior cervical sympathetic ganglia develop manifestations similar to those seen in Parry-Romberg syndrome in humans (Resende et al., 1991).

A number of so called neuro-cutaneous diseases show manifestation in the face (Little et al., 2015). For example, pigmentation defects such as Nevus of Ota or vitiligo, which involve loss or overproduction of pigmentation in specific locations, are often associated with changes in facial neuroanatomy (Nelhaus, 1970). In the case of Nevus of Ota the affected locations correspond to areas supplied by branches of the trigeminal nerve (Jovovic-Dagovic et al., 2007; Trufant et al., 2009). Further, the development of vitiligo has been related to dysfunctions in the sympathetic nervous system (Wu et al., 2000; van Geel et al., 2012), since certain cases of segmental vitiligo were found to develop after sympathectomy in patients (Lerner et al., 1966). Parry-Romberg syndrome has also been associated with sympathetic nerves (Janowska et al., 2013), and in addition both Parry-Romberg syndrome and vitiligo are linked to immune system dysfunctions and local inflammatory processes (Creus et al., 1994; van Geel et al., 2012). Interestingly, several studies have already pointed out at a connection between sympathetic nerves and local inflammation. This might play a role in the autoimmune component of vitiligo, with a malfunction of the primary beta-adrenoceptor signaling system (Wu et al., 2000). Furthermore, adrenergic compounds that are released from sympathetic nerves may serve as initiating or facilitating factors for numerous Th1-sustained inflammatory skin diseases (Manni and Maestroni, 2008).

Yet another nerve-associated tissue defect is Neurofibromatosis, which manifests itself through benign tumors and pigmentation spots that originate from peripheral nerves (Abramowicz and Gos, 2014). This is somewhat similar to a malicious contagious facial Schwannoma cancer, which almost eradicated the population of Tasmanian devils (*Sarcophilus harrisii*; Murchison et al., 2010).

As seen from above, the spectrum and severity of craniofacial anomalies involving nerves differ widely, but all cause some degree of functional physiological impairment. If these embryonic defects are too extensive, they can drastically reduce fetus survival rate (Sperber et al., 2010). It may also be added that distortions due to congenital disease may not only gravely impair obvious physiological craniofacial activities such as e.g. feeding, breathing, hearing and vision, but also cause dysfunctions in complex social communications (Jack and Schyns, 2015), and in this way severely reduce the quality of life.

### Further reading:

1. Non-canonical functions of the peripheral nerve (Kaucká and Adameyko, 2014).
2. Nerve dependence in tissue, organ, and appendage regeneration (Kumar and Brockes, 2012).
3. Roles of innervation in developing and regenerating orofacial tissues (Pagella et al., 2014).

## The peripheral nerve as a provider of necessary cell types in cranial development

**Figure 5:**
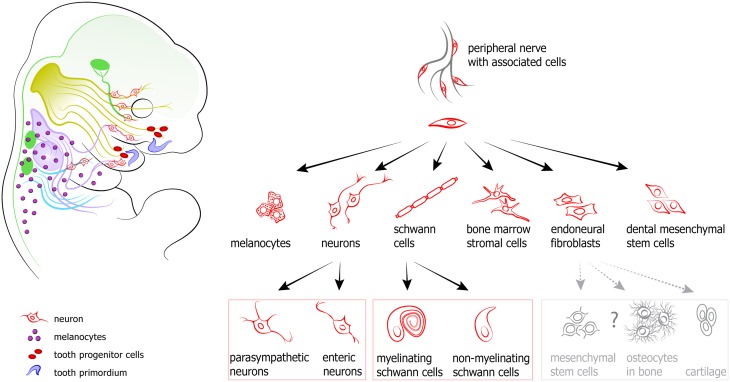
**Peripheral nerves host and transport neural crest-derived associated glial cells that give rise to a number of cell types in developing body**. **Left:** examples and position of nerve-derived cell types. A significant number of melanocytes (purple circles on a scheme) in a head-neck region is produced from recruited Schwann cell precursors. Additionally, some mesenchymal progenitors in developing teeth (shown as red ellipses) are also recruited from the peripheral nerves. At the same time all parasympathetic neurons in the head are derived from nerve-associated cells. **Right:** the spectrum of currently confirmed nerve-derived cell types.

The timing of the activation of different cellular sources for coordinated cranial development is tightly controlled. It enables the impressive evolutionary flexibility of head development that underlies the heterochrony in multiple vertebrate species. Recently, a novel cellular source has emerged as a key factor in the formation of a complete and functional head: the nerve-associated neural crest-derived cells (Schwann cell precursors, SCPs). The latest discoveries in the field demonstrate that these nerve-dwelling glial cells can produce other cell types, and through this participate in developmental mechanisms that shape and integrate the cranial compartment. Peripheral nerves can be viewed as navigating routes and a stem cell niche from which various cell types, including myelinating and non-myelinating Schwann cells, endoneural fibroblasts, parasympathetic neurons, enteric neurons, bone marrow mesenchymal cells and melanocytes originate at different time points and in various locations (Joseph et al., 2004; Adameyko et al., 2009, 2012; Budi et al., 2011; Laranjeira et al., 2011; Nitzan et al., 2013; Dyachuk et al., 2014; Espinosa-Medina et al., 2014; Isern et al., 2014; Uesaka et al., 2015) (see Figure 5).

### Cranial parasympathetic neurons originate from nerve-associated embryonic glial progenitors

The nerve cells of the parasympathetic ganglia of the head send their axons to various adjacent structures that are of fundamental importance for normal homeostasis. These structures include salivary and lacrimal glands as well as ciliary muscles of the eye. It has long been assumed that the parasympathetic neurons of the head, similar to the sensory ganglia of the cranial nerves, are derived from migrating specified NCCs. This has remained somewhat of an enigma, since parasympathetic gangliogenesis does not occur in the developing embryo until time points when the short pulse of neural crest migration already has ceased. Remarkably, it was recently found that these neurons actually originate not from waves of migrating NCCs, but instead from cells that initially travel to their target areas on outgrowing nerve fibers. In most cases these precursors follow the preganglionic axon route to the target locations. At the sites of the different future ganglia, among them the ciliary, the submandibular, the sphenopalatine and the otic ganglia, SCPs detach and change fate into parasympathetic nerve cells (Dyachuk et al., 2014; Espinosa-Medina et al., 2014). This conversion from glial to neuronal phenotype seems to be triggered by a local induction of ASCL1, which in turn may be influenced by local secretion of BMPs and/or Wnts (Müller and Rohrer, 2002; Knosp et al., 2015). This both unanticipated and sophisticated process shows how the developing nervous system, using an intrinsic machinery, can both transport necessary nerve cells as precursors to the proper target site using matrices laid out by the preganglionic axons, and, when they subsequently have changed phenotype, connect these postganglionic neurons to the same preganglionic axons that they previously trekked on. Obviously, this raises a number of challenging questions related to the extremely detailed temporal coordination of gene expression that underlies this complex mechanism (Lleras-Forero and Streit, 2012). What signals induce the genes that specify parasympathetic neuronal fate in local SCPs in extremely restricted locations in the head? Are these signals coordinated with the genetic networks that induce the actual target organ formation? Do the peripheral neural components control the development of craniofacial elements?

To summarize, the development of parasympathetic ganglia requires a late progenitor cell source that can ensure a neural crest type of neurogenesis long after neural crest migration is over. This is solved by SCPs. The concept of using SCPs from presynaptic nerves facilitates navigation, and eventually establishes and warrants synaptic connectivity far away from the original neural crest source (Adameyko and Ernfors, 2014; Ivashkin et al., 2014).

### Cranial pigment cells, melanocytes, are derived in part from nerve-associated schwann cell precursors

Melanocytes, the pigment cells, are important not only for the color of the eyes or pigmentation of hairs and skin in the head, but they are also essential for hearing. Melanocytes residing in the inner ear are indeed an essential component of the integral inner ear environment that is necessary for survival of sensory hair cells. The melanocyte as a cell type is essential for proper cochlea development, since mutant mice without melanocytes develop impaired auditory structures (Hozumi et al., 2012; Ni et al., 2013). At the same time, melanin does not seem to be important for the sensory function of the ear (Tachibana, 1999). Additionally, melanocytes are abundant in brain meninges (Painter et al., 2000), but their function there is still unclear. All these extracutaneous cranial compartment melanocytes might be important points of origin for malignant melanomas (Hussein, 2008). Recently, a new origin of melanocytes has been revealed in a number of species, in particular in mouse, chick, and fish (Adameyko et al., 2009, 2012; Budi et al., 2011; Dooley et al., 2013; Nitzan et al., 2013). In all of those cases melanocytes have been produced not only by NCCs directly, but also from nerve-associated glial cells: SCPs. In the mouse, the first melanoblasts appear inside of the IXth and Xth cranial nerves in the E9.5 embryo. These melanoblasts migrate outwards to subepithelial spaces 1 day later to extensively proliferate and populate all adjacent areas. Other facial nerves produce melanocytes as well. Edn3 and Kit ligand are essential signals for propagating these nerve-derived melanocytes in the head and neck (see Adameyko et al., 2012).

The close association of melanocyte progenitors with nerves may provide a logic for neural crest evolution (Ivashkin and Adameyko, 2013) and explain how melanocytes can find routes to the iris and interior of the cochlea, as well as to other locations deep inside of the head (Adameyko and Lallemend, 2010).

### Nerve-derived mesenchymal stem cells participate in tooth development and repair

Peripheral ganglia and nerves together with associated glia, to a large extent derived from migratory NCCs, develop and target all parts of the head, starting from relatively early embryonic stages. In mice the tooth pulp receives its nerve supply very late (P3-4) in comparison to the surrounding mesenchymal tissue, which is densely innervated already early (E12.5) when tooth placodes are formed (Fried et al., 2000). There has been substantial interest in the potential of SCPs to act as multipotent progenitor cells of the PNS. Considering the significant inherent plasticity of the niche containing SCPs, it could constitute a cellular reservoir during tooth organogenesis. Technically, it has been a daunting task to address this issue, since both ectomesenchymal cells and glial cells of the peripheral nerve are of neural crest origin. However, mouse strains that allow for selective genetic labeling of peripheral glial cells have provided a tool for this purpose (Adameyko et al., 2009, 2012). Results from studies on PLP-CreERT2 and Sox10-CreERT2 mice have demonstrated that SCP-derived cells in the surrounding area of developing teeth contribute with pulp cells as well as odontoblasts in a clonal pattern (Kaukua et al., 2014). This mechanism continues to operate also in the adult, as determined in the continuously growing mouse incisor. Furthermore, the generation of SCP-derived progeny of dental MSCs and odontoblasts is impaired after denervation (Kaukua et al., 2014).

It is interesting to consider the novel model of glial-assisted tooth development and growth (Kaukua et al., 2014) in light of earlier proposals that the tooth, from an evolutionary aspect, is a primary sensory organ. According to this view, dentin is a tissue that developed in order to protect and enhance the sensitivity of a pre-historical sensory organ (Gans and Northcutt, 1983). This fits well with the fact that matrix-producing odontoblasts are intimately associated with sensory nerve endings that transduce somatic signals (experienced as pain) in the mammalian tooth (Magloire et al., 2009, 2010). Odontoblasts could then be seen as adapted terminal glial cells, similar to the modified Schwann cells that enfold nerve fibers in sensory end-organs in the skin such as e.g. Meissner's and Pacinian corpuscles.

### Peripheral glial cells might mediate important signals during development and regeneration in the head

The impact of the peripheral nervous system on wound healing and tissue regeneration in mammalian head has been under debate. Wounded pinna, the outer ear, serves as a model system to study regeneration in mammals. Mice from MRL/MpJ strain are capable of enhanced regeneration of the ear wounds as compared to the other strains, and such regenerative capacity is enhanced by the presence of the nerves densely surrounding regenerating tissue. Denervation of the ear leads to obliteration of regenerative capacity in this model system (Buckley et al., 2012).

In addition to this, genetic lineage tracing and clonal analysis of individual cells in denervated mouse limb tissues during regeneration demonstrated that cellular turnover, and differentiation from stem/progenitor cells remain functionally independent of nerve and nerve-derived factors. However, regenerated digit tips displayed patterning defects in bone and nail matrix. Interestingly, these nerve-depe ndent phenotypes mimic clinical observations of patients with nerve damage after spinal cord injury (Rinkevich et al., 2014). Recent discoveries from Jeremy Brockes' laboratory highlight the specific role of nerve-associated glial cells in regenerative blastema formation during regeneration of salamander tissues (Kumar et al., 2007; Kumar and Brockes, 2012). These studies demonstrated that the nerve-associated peripheral glial cells release nAG—anterograde signaling protein that initiates de-differentiation of multiple cell types in a wounded bodypart of salamander. Therefore, nerve-associated cells might be important integral components of the nerve that are responsible for the crosstalk between the nerve and innervated tissue during regeneration and healing. This is further supported by cellular and molecular mechanisms of neuro-vascular alignment, where embryonic peripheral glial cells release the signals that pattern and rebuild the vessels into arteries and arterioles during development of vertebrate embryos (Li et al., 2013).

The facts that nerves seem to be involved in the regeneration of injured mineralized mammalian limb tissue, and that nerve fiber glia is a reservoir of dental MSCs in the adult tooth, at least in the mouse incisor, could infer that nerve-borne cells might be mobilized after injury and contribute to regenerative and reparative events. Examinations in damaged mandibular incisors of adult PLP-CreERT2/R26YFP confirmed that SCP-derived cells were gathered at the site of damage, many of which had attained the characteristics of matrix-secreting odontoblasts. Pericytes, which have previously been shown to generate odontoblasts after injury (Feng et al., 2011), were excluded as an intermediate cell type in this case (Kaukua et al., 2014).

### Aligned pericytes and nerves: strategies converge

The vasculature of the dental pulp follows the same routes as the nerves, as in majority of locations in the head. A layer of loose connective tissue surrounds many arteries and nerves, forming a neurovascular bundle. This bundle constitutes a niche for MSCs that participate in both homeostasis and injury repair in teeth (Zhao et al., 2014). Previously, it has been shown that SCPs of peripheral nerves secrete CXCL12 that attracts the endothelial cells to align adjacent to the nerves during development (Li et al., 2013). This demonstrates the presence of a sophisticated system where nerves direct the development of an accompanying primary vessel network. A continued nerve-vessel crosstalk might also influence the neurovascular tissue homeostasis in the adult. This is indicated by studies of the development of the arterial innervation. Thus, in addition to vascular tone, sympathetic nerves also influence arterial maturation and growth through VEGF-dependent neurovascular synapses (Mukouyama et al., 2005).

Furthermore, the vasculature along peripheral nerves contains pericytes - contractile multifunctional cells that wrap around the endothelial cells of capillaries and venules within the vascular basement membrane (Sims, 1986). Cephalic pericytes in the forebrain seem to be neural crest-derived, as demonstrated in chick-quail chimeras (Etchevers et al., 2001; Korn et al., 2002) and in mice (Heglind et al., 2005). Pericytes in the other parts of the body are believed to be of mesodermal origin (Mills et al., 2013). It has been suggested that pericytes (also called adventitial or Rouget cells) may represent mesenchymal stem or progenitor cells (Crisan et al., 2008), since they can differentiate into a variety of MSC cell types, such as fibroblasts, chondroblasts, osteoblasts, odontoblasts, adipocytes, vascular smooth muscle cells, and myointimal cells (Díaz-Flores et al., 2006; Armulik et al., 2011; Feng et al., 2011). Some pericytes express markers characteristic for stem cells, such as Sca1 (Brachvogel et al., 2005) and STRO-1. STRO-1 is an early common marker for MSCs (Yoshiba et al., 2012). It has been demonstrated that upon tissue damage pericytes leave the perivascular space and generate myofibroblasts, thus playing a central role in organ fibrosis after injury. Ablating these cells ameliorates fibrosis and rescues organ function (Kramann et al., 2015). Moreover, a specific pericyte subtype gives rise to scar-forming stromal cells in CNS after the injury (Göritz et al., 2011). Ablation of these cells results in failure to seal the damaged CNS. In the continuously growing mouse incisor, pericytes play an important role in regeneration after the damage: they manage to leave the vessels and generate matrix-producing odontoblasts (Feng et al., 2011). Additionally, pericytes serve as a continuous cell source for matrix-laying cells at the very tip of the incisor. This region bears the maximum of the bite load, and wears out on a daily basis. In particular, pericytes generate streams of cells that obliterate the pulp cavity with hard matrix at the cutting surface of the tooth (Pang et al., 2015). Thus, pericytes play an important role in vascular development and homeostasis, are sources of fibrogenic cells in pathological situations, and may also serve as a reservoir of stem or progenitor cells for adult tissue repair in the cranial compartment.

The system of recruiting pericytes from vessels in development and regeneration strongly resembles the mechanism whereby peripheral glial cells are mobilized from nerves in the same processes. Here, we observe converging strategies that provide the developing or regenerating tissues with necessary progenitor cells. Thus, since craniofacial nerves and vessels usually are intimately associated, specific molecular signals may simultaneously engage both glial cells and pericytes for developmental and regenerative purposes in the head.

### Further reading:

1. A paradigm shift in neurobiology: peripheral nerves deliver cellular material and control development (Ivashkin et al., 2014).
2. Nerves transport stem-like cells generating parasympathetic neurons (Adameyko and Ernfors, 2014).
3. Progenitors of the protochordate ocellus as an evolutionary origin of the neural crest (Ivashkin and Adameyko, 2013).
4. Glial versus melanocyte cell fate choice: Schwann cell precursors as a cellular origin of melanocytes (Adameyko and Lallemend, 2010).
5. A perivascular origin for mesenchymal stem cells in multiple human organs (Crisan et al., 2008).

## Summary and perspectives

Recently, a growing amount of data has provided a deeper insight into how various cellular sources are coordinated and integrated during craniofacial development. The cranial nervous system, including the developing anterior neuroectoderm, brain, neurogenic placodes and peripheral nerves has appeared as an essential element in a number of regulatory interactions that result in a fully functional complex craniofacial organ.

Novel data on interactions between neurogenic placode-derived organs and their encasing cartilage or bone has improved the understanding of developmental coordination in a head. These processes are of utmost importance from an evolutionary perspective, since they will ensure the best functional outcome of cranial placode-derived sensory organs. A recently revealed brain-to-face interaction has highlighted a new degree of contribution of nervous system in the creation of the head. A plethora of functions of peripheral nerves in development was recently revealed. These nerves appear to serve as morphogenetic signal-releasing conduits as well as niches for multipotent glial cells that can be transformed into a spectrum of differentiated cell types in developing head. The importance of peripheral nerves in the cranial compartment is highlighted by a number of congenital or acquired pathologies associated with PNS development.

Taken together, it seems that neural components have co-evolved in tight coordination with other tissues and cell types in a head. Such a high degree of reciprocal influence should in principle increase developmental and evolutionary plasticity, and lead to optimal design and functional success. We anticipate that novel molecular interactions that aim to integrate various cell and tissue types soon will be discovered in craniofacial development. This will undoubtedly improve our fundamental understanding of coordinated growth strategies in this region of the body—an understanding that will benefit and empower the field of craniofacial regeneration.

## Author contributions

Both authors listed, have made substantial, direct and intellectual contribution to the work, and approved it for publication.

### Conflict of interest statement

The authors declare that the research was conducted in the absence of any commercial or financial relationships that could be construed as a potential conflict of interest.
